# Antisolvent 3D Printing of Gene-Activated Scaffolds for Bone Regeneration

**DOI:** 10.3390/ijms252413300

**Published:** 2024-12-11

**Authors:** Andrey Vyacheslavovich Vasilyev, Irina Alekseevna Nedorubova, Viktoria Olegovna Chernomyrdina, Anastasiia Yurevna Meglei, Viktoriia Pavlovna Basina, Anton Vladimirovich Mironov, Valeriya Sergeevna Kuznetsova, Victoria Alexandrovna Sinelnikova, Olga Anatolievna Mironova, Ekaterina Maksimovna Trifanova, Igor Ivanovich Babichenko, Vladimir Karpovich Popov, Anatoly Alekseevich Kulakov, Dmitry Vadimovich Goldshtein, Tatiana Borisovna Bukharova

**Affiliations:** 1 Central Research Institute of Dentistry and Maxillofacial Surgery, 119021 Moscow, Russia; irina0140@gmail.com (I.A.N.); victoria-mok@yandex.ru (V.O.C.); an.megley95@yandex.ru (A.Y.M.); scftlab@gmail.com (A.V.M.); tilia7@yandex.ru (V.S.K.); sinelnikova_va@cniis.ru (V.A.S.); mironova.o.a@yandex.ru (O.A.M.); katikin@mail.ru (E.M.T.); babichenko_ii@cniis.ru (I.I.B.); bukharova-rmt@yandex.ru (T.B.B.); 2 Research Centre for Medical Genetics, 115478 Moscow, Russia; vika.basina12@gmail.com (V.P.B.);; 3 NRC «Kurchatov Institute», 123182 Moscow, Russia; vladikarpopov@gmail.com

**Keywords:** 3D printing, adenoviral vector, BMP-2, bone regeneration

## Abstract

The use of 3D-printed gene-activated bone grafts represents a highly promising approach in the fields of dentistry and orthopedics. Bioresorbable poly-lactic-co-glycolic acid (PLGA) scaffolds, infused with adenoviral constructs that carry osteoinductive factor genes, may provide an effective alternative to existing bone grafts for the reconstruction of extensive bone defects. This study aims to develop and investigate the properties of 3D scaffolds composed of PLGA and adenoviral constructs carrying the BMP2 gene (Ad-BMP2), both in vitro and in vivo. The elastic modulus of the disk-shaped PLGA scaffolds created using a specialized 3D printer was determined by compressive testing in both axial and radial directions. In vitro cytocompatibility was assessed using adipose-derived stem cells (ADSCs). The ability of Ad-BMP2 to transduce cells was evaluated. The osteoinductive and biocompatible properties of the scaffolds were also assessed in vivo. The Young’s modulus of the 3D-printed PLGA scaffolds exhibited comparable values in both axial and radial compression directions, measuring 3.4 ± 0.7 MPa for axial and 3.17 ± 1.4 MPa for radial compression. The scaffolds promoted cell adhesion and had no cytotoxic effect on ADSCs. Ad-BMP2 successfully transduced the cells and induced osteogenic differentiation in vitro. In vivo studies demonstrated that the 3D-printed PLGA scaffolds had osteoinductive properties, promoting bone formation within the scaffold filaments as well as at the center of a critical calvarial bone defect.

## 1. Introduction

Modern advances in regenerative medicine are opening up new horizons for the repair of damaged tissues and organs. In dentistry and orthopedics, the problem of restoring extensive bone defects is important because it can significantly affect the quality of life of patients [1]. Current clinical solutions often rely on standardized shapes of bone scaffolds that may not adequately address unique defect characteristics such as size, shape and location. In this context, 3D-printing technologies represent a promising approach [2,3,4]. It helps to create personalized and biocompatible scaffolds that closely mimic the anatomy of the affected bone area, improving the likelihood of successful integration and healing [5,6].

As a biocompatible and biodegradable polymer, polylactic-co-glycolic acid (PLGA) has excellent mechanical properties and can be chemically modified to change its properties. This makes it an ideal material for bone tissue regeneration [7]. PLGA scaffolds maintain their structural integrity during the early stages of bone repair, gradually degrading into non-toxic by-products without the need for secondary surgical intervention [8]. The use of 3D printing enables fine-tuning of the scaffold architecture, which helps to improve cell adhesion and vessel ingrowth [9]. However, PLGA may not result in a significant bone healing response and may need to be combined with an osteoinductive component for optimal bone regeneration [7].

Obtaining biologically activated polyether-based matrices is a challenging task, primarily due to the difficulty of processing under certain conditions. One major drawback of traditional approaches to producing polyester-based scaffolds, such as PLGA, is the need for non-physiological temperatures (60 °C or higher) or the use of toxic organic solvents, while resulting scaffolds have low porosity [10]. This makes it difficult to create scaffolds containing thermolabile components, such as growth factors or drugs [11]. An alternative approach to avoiding these disadvantages is to use a biocompatible PLGA solvent, such as tetraglycol, which is suitable for water-assisted polymer precipitation or “antisolvent structure formation”. The tetraglycol can easily be extracted from the PLGA solution by water, inducing rapid phase separation and creating a scaffold with a different type of microstructure. These principles are based on anti-solvent 3D printing, a new approach to free-form fabrication that is performed under normal conditions with biocompatible materials [12].

While the clinical use of osteoinductive bone morphogenetic protein BMP-2 has shown positive results in promoting bone healing, several complications have been reported following its administration, including adverse effects related to inflammation and ectopic bone formation [13].

The disadvantages of using this protein have led to the development of alternative treatment methods in order to reduce the risk of potential side effects. These methods involve the use of genetic constructs containing the BMP2 gene, which are impregnated into scaffolds. These gene-activated scaffolds then release vectors that stimulate protein synthesis in resident cells, leading to regenerative processes in bone tissue [14].

Viral vectors, in contrast to non-viral methods, provide more effective gene delivery, especially in vivo, and promote prolonged gene expression compared to non-viral constructs, allowing for the creation of gene-activated scaffolds with a lower concentration of vectors to achieve a therapeutic effect [15,16]. Adenoviral constructs with a high transduction efficiency and large packaging capacity are promising for clinical use [17]. The low risk of insertional mutagenesis and, consequently, tumor development, as well as the transient nature of transduction, make them attractive for gene therapy [18,19]. The disadvantage of using adenoviral constructs is their immunogenicity, which reduces the duration of transgene expression. However, even short-term secretion of the target protein will cause an osteogenic effect, while also minimizing potential undesirable side effects on bone tissue, such as hyperostosis and heterotopic osteogenesis [20,21].

The aim of this work is to investigate the properties of 3D-printed scaffolds based on polylactide-co-glycolide and adenoviral constructs carrying the *BMP2* gene in vitro and in vivo and to provide a perspective for their use in dentistry and orthopedics.

## 2. Results

### 2.1. Scaffold Structure

**Figure 1:**
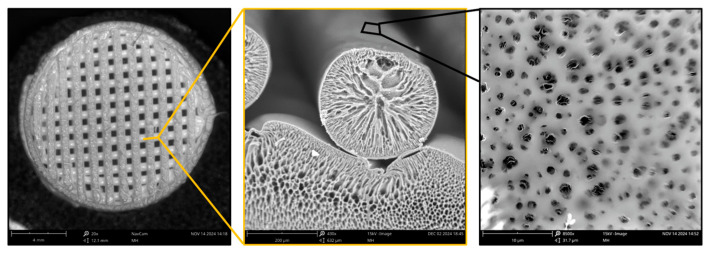
SEM images of the filament structure and internal structure of PLG scaffold formed by anti-solvent 3D printing.

The internal structure of the filaments exhibited a porous architecture with a distinct radial orientation (see Figure 1). This structural formation can be attributed to the development of vigorous countercurrent flows of the polymer solution and the antisolvent during the solidification process of the polymer. Additionally, the surface of the filaments displayed a porous configuration; however, the pore size was significantly smaller by an order of magnitude. This unique morphology facilitated both the impregnation and subsequent release of the adenoviral constructs.

### 2.2. Mechanical Properties

**Figure 2:**
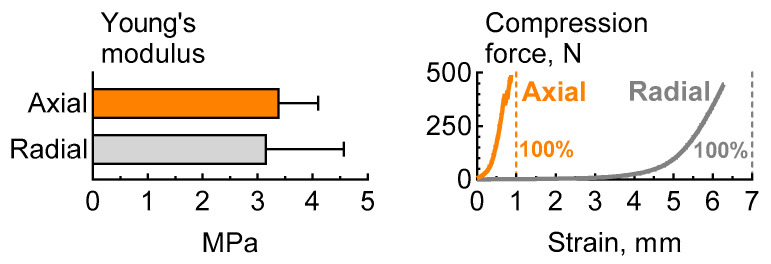
Means of Young’s modulus at radial and axial compression of PLGA scaffold.

The elastic modulus measured at a 10% deformation of the PLGA scaffold samples was nearly identical in both loading directions. The axial compressive modulus was (3.4 ± 0.7) MPa and did not statistically differ from the radial compressive modulus, which was (3.17 ± 1.4) MPa (Figure 2). There was no occurrence of sample failure or pore collapse, as evidenced by the proportional limit, when subjected to compression up to 30%. Similar data regarding the elastic properties suggest that the interconnection of the filaments, which provides the macrostructure, is sufficiently stable under load, while the strength primarily depends on the porosity of the filament itself, that is, the microstructure.

### 2.3. In Vitro Study

#### 2.3.1. Evaluation of the Cytocompatibility of PLGA+Ad-BMP2 Scaffolds

**Figure 3:**
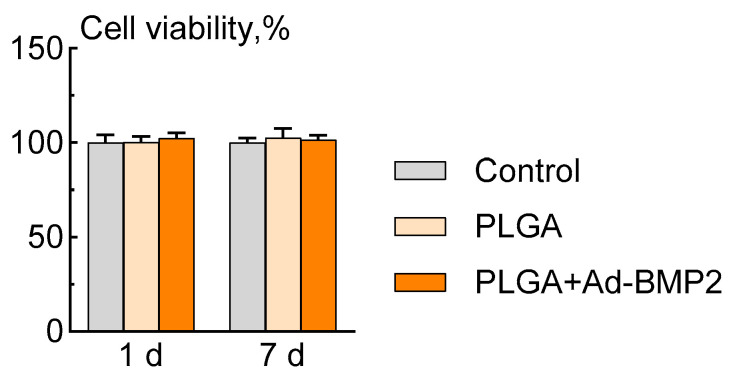
Viability of ADSCs at 1 and 7 days after incubation with scaffolds, MTT test.

After 1 and 7 days, there were no statistically significant differences in the relative viability of ADSCs after incubation with the tested PLGA scaffolds compared to control samples (Figure 3).

**Figure 4:**
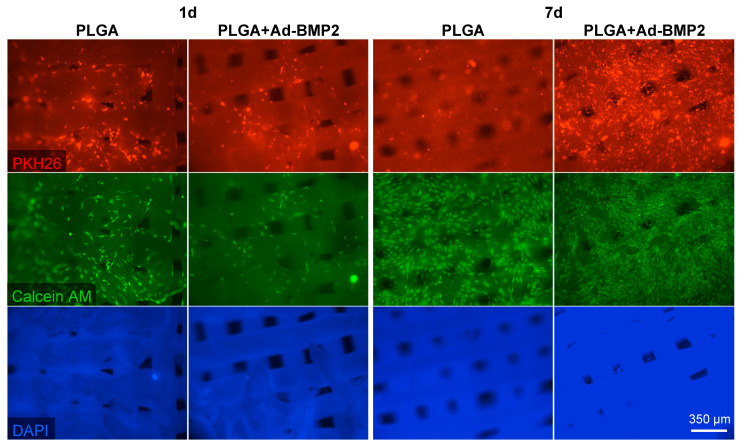
Adhesion of ADSCs on the surface of scaffolds. Fluorescence microscopy.

It was observed that ADSCs adhered to the matrix surfaces within 24 h. By day 7, the density of live cells, stained with Calcein AM, had significantly increased. No dead cells, stained with DAPI, were detected (Figure 4). Thus, the scaffolds exhibit cytocompatibility, and the incorporation of adenoviral constructs carrying the *BMP2* gene into the scaffolds does not exert toxic effects on the cells.

#### 2.3.2. Evaluation of the Transduction Ability of Ad-BMP2 Incorporated into PLGA-Based 3D Scaffolds

Adenoviral constructs containing the *BMP2* gene, released from PLGA-based 3D matrices, demonstrated effective transduction capabilities. After 7 days of incubation of ADSCs in the presence of the PLGA+Ad-BMP2, expression of the target gene and protein BMP-2 was observed.

**Figure 5:**
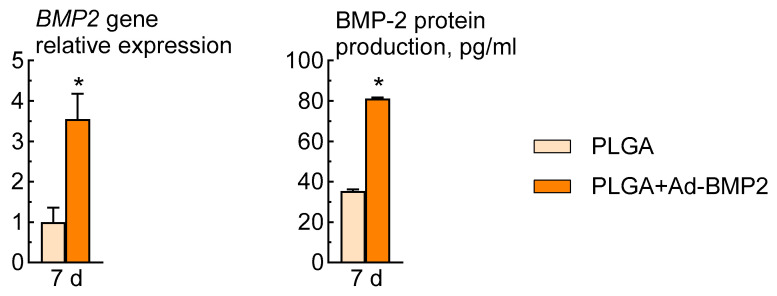
Transduction efficiency of Ad-BMP2 incorporated into PLGA-based 3D scaffolds. Relative expression of osteogenic marker genes RT-PCR; osteogenic marker proteins production, ELISA. * *p* < 0.001.

The expression level of the target gene *BMP2* increased by 3.5 ± 0.6 compared to the PLGA-based 3D scaffolds without adenoviral constructs. Furthermore, the production of BMP-2 protein in cells cultured in the presence of PLGA+Ad-BMP2 scaffolds also increased 2.3 times compared to the control group, reaching a concentration of 81 ± 1 pg/mL (Figure 5).

#### 2.3.3. Osteogenic Differentiation of ADSCs Incubated with PLGA-Based 3D Scaffolds Impregnated with Ad-BMP2

The 3D-printed scaffolds impregnated with PLGA+Ad-BMP2 were able to induce osteogenic differentiation of ADSCs. After 7 and 14 days, there was a statistically significant increase in *Alpl*, *Bglap* and *Spp1* gene expression in ADSCs compared to cells cultured in the presence of a PLGA scaffold and a control group without material.

**Figure 6:**
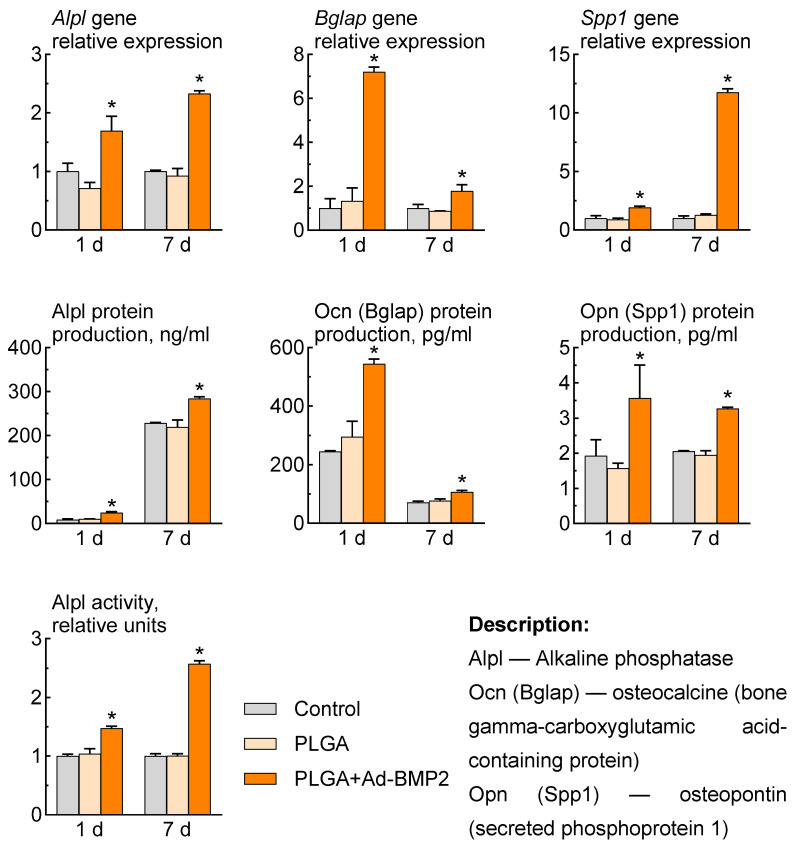
Osteogenic differentiation of ADSCs after incubation with scaffolds: relative expression of osteogenic marker genes, RT-PCR; osteogenic marker proteins production, ELISA; enzyme activity, spectrophotometry. * *p* < 0.001 (relative to control).

When ADSCs were incubated with PLGA+Ad-BMP2, we also found an increase in the production of the corresponding proteins and an increase in alkaline phosphatase activity compared to the control groups (Figure 6).

#### 2.3.4. Histological Study

**Figure 7:**
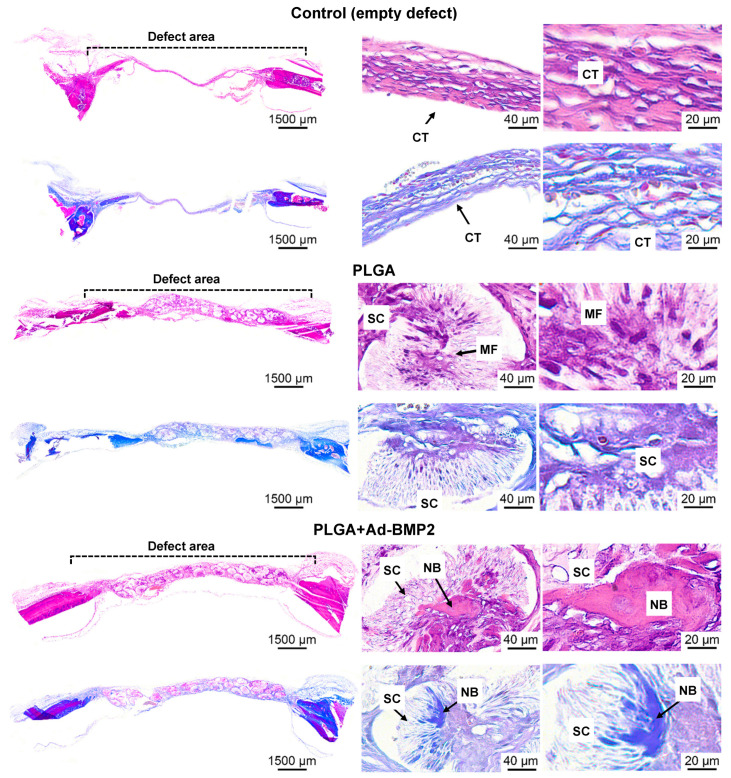
Histological study of a critical-size rat calvarial bone defect after implantation; H&E or Masson’s trichrome staining. CT—connective tissue, MF—macrophage, NB—new bone, SC—scaffold.

The osteoinductive and biocompatible properties were investigated by implantation into the critical defect of rat calvarial bone. After 56 days, bone tissue ingrowth was observed within or around the filaments of PLGA+Ad-BMP2 scaffolds. After implantation of plain PLGA scaffold connective tissue, macrophages migrated inside the filaments, and no newly formed bone tissue was detected inside the material; it only grew from the edges of the defect. The empty defect (‘control’) was overgrown with connective tissue in the center and there were also foci of newly formed bone tissue at the periphery in contact with the rat bone (Figure 7).

**Figure 8:**
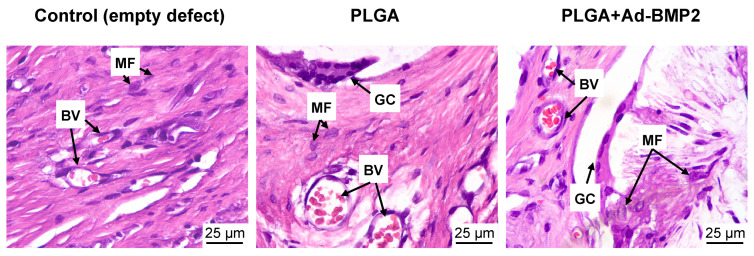
Connective tissue and immune cells in the area of the defect and between the scaffold filaments. H&E. MF—macrophage, GC—giant cell, BV—blood vessel.

The resorption of the scaffold’s filaments occurred due to macrophages and foreign-body giant cell that grew into the center of the filaments. Isolated or small clusters of lymphocytes were present in individual fields of view. Overall, the results were consistent with the resorption of lactic acid copolymer materials, and no significant inflammation was observed (Figure 8).

#### 2.3.5. Histomorphometric and Immunohistochemical Analysis

**Figure 9:**
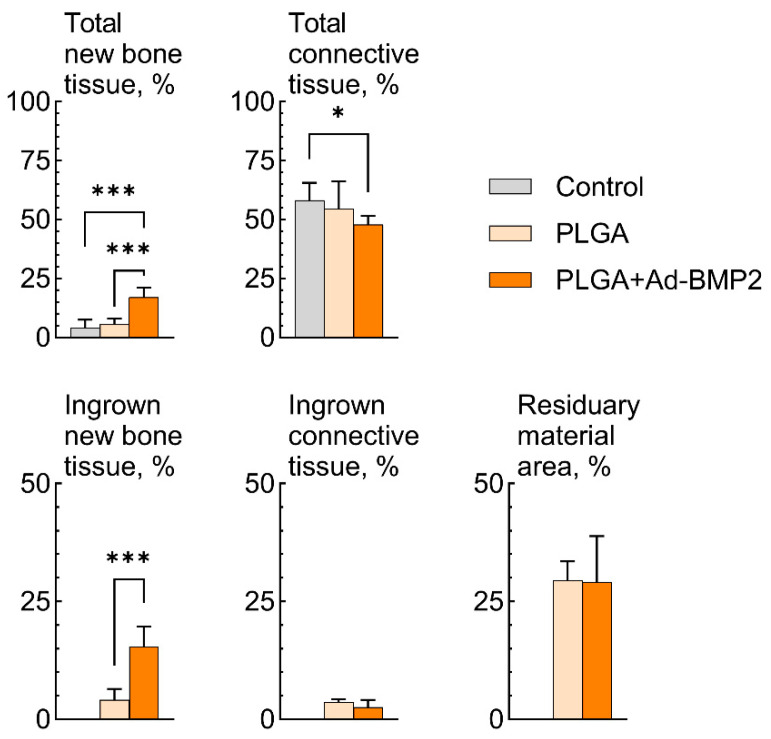
Results of morphometrical study of a critical-size rat calvarial bone defect after implantation in control, PLGA, PLGA+Ad-BMP2 groups. * 0.05 > *p* > 0.01; *** *p* < 0.001.

The results of morphometric study showed that implantation of PLGA-Ad+BMP2 scaffolds resulted in formation of bone tissue in a significantly larger volume in comparison with the empty defect and implantation of PLGA scaffolds. Moreover, stimulated neoosteogenesis predominantly occurred in the space inside the scaffold (Figure 9).

The volume of PLGA scaffolds’ resorption with and without adenovirus was not statistically significantly different. The presence of the adenoviral vector did not induce the macrophage response in a distinct way and did not change the dynamics of material resorption at 28 days.

In an immunohistochemical study, staining for osteopontin (Opn) was observed in the pore space of 3D-printed scaffolds with Ad-BMP2, which was not detected in the control groups.

**Figure 10:**
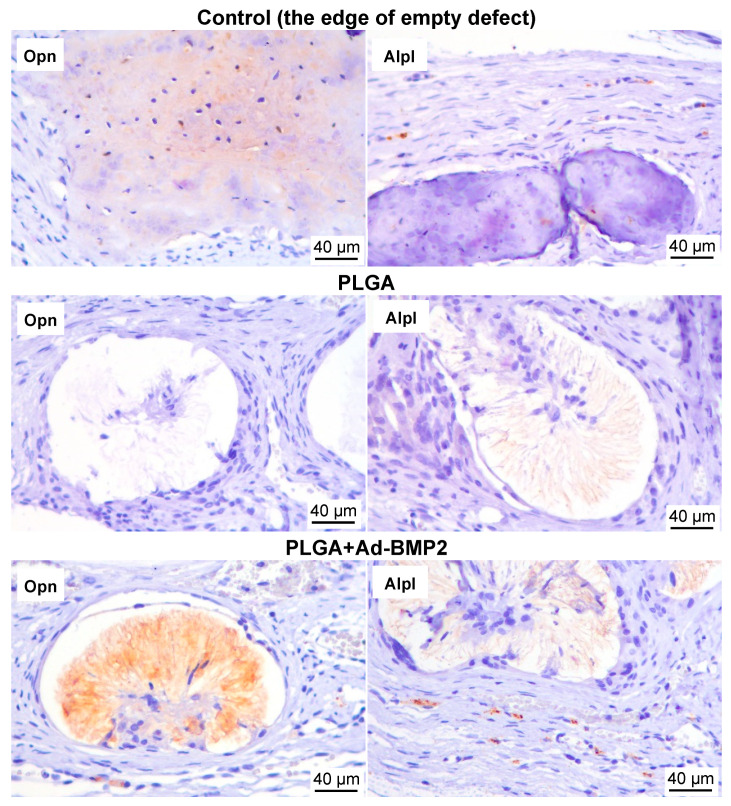
Immunohistochemical study of regenerate in the area of critical rat calvarial defect after implantation. Staining for osteopontin (Opn) and alkaline phosphatase (Alpl) (brown).

The exception to this was the area near the edge of the defect, where staining for osteopontin was observed within the matrix of newly formed bone tissue (Figure 10). Alkaline phosphatase expression was mainly detected adjacent to the newly formed bone tissue at the edge of the defect and only within the space between filaments of PLGA+Ad-BMP2 (Figure 10). Thus, immunohistochemical staining confirmed osteoinductive properties of the scaffolds with Ad-BMP2.

## 3. Discussion

The present study demonstrated the feasibility of producing a 3D-printed gene-activated bone tissue graft based on poly-lactic-co-glycolic acid and an adenoviral vector carrying the *BMP2* gene.

The primary advantages of the developed scaffold are its biological properties. A specially developed 3D-printing technique ensures the preservation of the activity of biologically active components by avoiding high temperatures or the presence of toxic solvents. As shown in previous studies, altering the parameters of poly(lactide-co-glycolide) deposition with an antisolvent leads to a slight change in its highly porous microstructure, which functions as a reservoir for biologically active substances, without affecting the biocompatibility of the resulting scaffolds [12,22,23].

In vitro analyses revealed cytocompatibility for PLGA+Ad-BMP2 scaffolds, coupled with demonstrated osteoinductive characteristics. ADSCs in the presence of the scaffold produced markers of osteogenic differentiation. Furthermore, the cytocompatibility and osteogenic properties were found to be comparable to other highly porous scaffolds activated by Ad-BMP2 or plasmids carrying the *BMP2* gene that we have previously studied [24,25].

Most of the research is aimed at developing hydrogel-based gene-activated scaffolds using 3D printing. Three-dimensional hydrogel scaffolds based on alginate and gelatin containing miR-29b caused osteoblastic differentiation of hMSCs and induced osteogenesis in vivo [26]. The effectiveness of 3D-printed alginate scaffolds impregnated with the plasmid containing the *BMP2* gene was demonstrated in stimulating new bone formation on the maternal side in a critical parietal bone defect model in rats [27]. In the study of Loozen et al. (2013), alginate-based scaffolds with MSCs transfected with BMP2 were produced using 3D bioprinting. This caused an increase in osteogenic markers under in vitro conditions. However, the implanted scaffolds did not show a significant osteogenic effect in vivo [28]. It is possible that the high rate of biodegradation of 3D hydrogel-based scaffolds, due to their low osteoconductivity, does not ensure efficient bone formation in vivo. The PLGA-based 3D scaffolds obtained in this study bioresorbed more slowly and remained at the site of the bone defect for 8 weeks (Figure 7). The developed gene-activated scaffolds PLGA+Ad-BMP2 supported cell migration and adhesion and had osteoinductive properties: they ensured the formation of bone tissue de novo within the scaffold structure, away from the margins of the existing bone defect. The efficacy of 3D-printed PLGA-based scaffolds loaded with cells transduced with a lentiviral vector containing the *BMP2* gene was also shown for the healing of a critical-sized femoral bone defect [29]. However, the need for cell culture and transduction steps, characteristic of the ex vivo approach, as well as the rapid elimination of transplanted cells, presents a significant disadvantage compared to our method of delivering genetic constructs directly to the defect area to transduce resident cells as they migrate into this area.

A comparison of the mechanical properties of the scaffolds we developed showed that the elastic modulus of 3D-printed PLGA scaffolds was higher than that of cartilage (250 kPa–3 MPa) but was approximately twice as low as that of bone tissue [30,31]. That is enough for the restoration of small and unloaded bone defects. However, to facilitate the reconstruction of diaphysis of tubular bones or fragments of the jaw, further technological development is necessary. It is imperative to recognize that reducing porosity may compromise osteoconductive properties.

We believe that future developments should focus on improving 3D-printing technology and studying the long-term effects of gene-activated scaffolds applications. A bone implant skeleton can be created without pores by using a tightly packed polymer with a high molecular weight. These filled structures can be produced using the methods described in this paper. We expect that this integrated approach will significantly improve the bone replacement process in the future.

In addition, this study has some limitations. While adenovirus vectors are generally considered relatively safe, further long-term research is needed to fully understand their potential immunogenic and other effects when used in vivo.

Therefore, the present study has demonstrated the properties of three-dimensional-printed gene-activated scaffolds using poly-lactic-co-glycolic acid and an adenoviral vector carrying the BMP2 gene. The results confirm the promising potential of this approach and provide a foundation for further development of protocols that could have clinical applications.

## 4. Materials and Methods

### 4.1. Materials

PLGA (Purasorb PDLG7507 Corbion PURAC Biochem, Gorinchem, The Netherlands) with a lactide/glycolide ratio of 75/25 and a characteristic viscosity of 0.7 dL/g was used. To obtain a 10 wt.% solution, 1 g of PLGA was mixed with 9 g of a non-toxic solvent tetraglycol (TG) (Sigma-Aldrich, St. Louis, MO, USA) using a magnetic stirrer (DLab FlatSpin, Beijing, China) for 2 days at room temperature.

### 4.2. Antisolvent Printing

**Figure 11:**
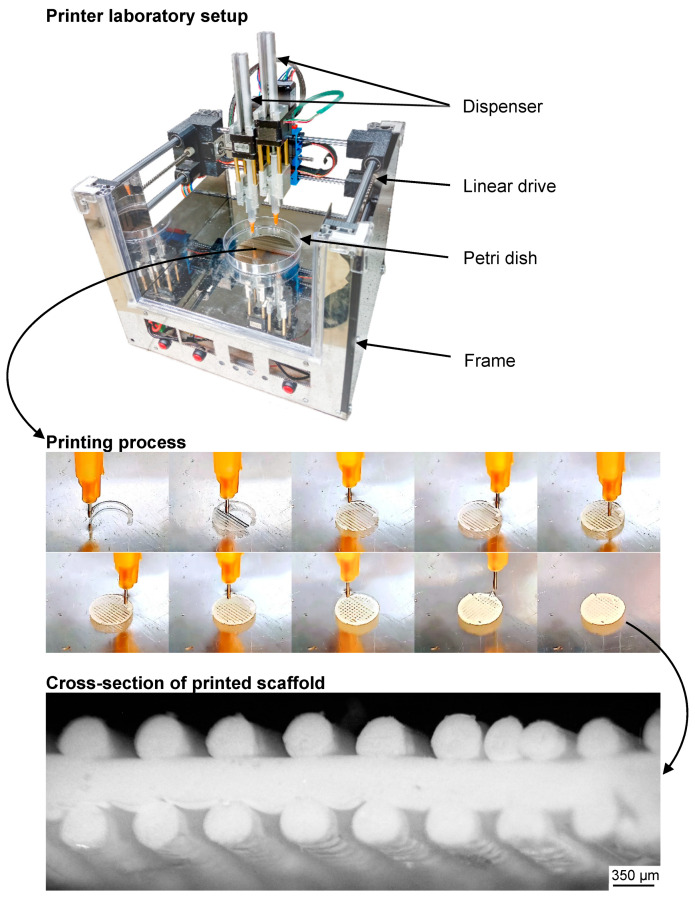
Laboratory 3D printer, process of layer-by-layer antisolvent printing and optical microscopy image of printed and cross-sectioned scaffold.

The formation of three-dimensional scaffolds was carried out on a specially designed double dispenser syringe 3D printer (NRC «Kurchatov Institute», Moscow, RF) (Figure 11). The basic principle of 3D printing was a precipitation of a preciously deposited PLGA-TG solution jet in water followed by structure hardening. The 3D printer was controlled by RepetierHost software v.2.2.4 (Hot-World GmbH & Co. KG, Willich, Germany) set a specific 25 °C temperature regime for printing, which was maintained by built-in thermoelectric converters (Peltier elements). A digital model of a 3-layer grind disk with a diameter of 7 mm and height of 1 mm was used as the printing object. The layer infill was set to 35%, the layer height was set at 300 µm and the linear printing speed was set to 2 mm/s.

In order to prepare the printing process, a 10% PLGA solution in TG was transferred into a 2 mL sterile disposable syringe. The solution inside the syringe was exposed to UV light for 10 min, under a lamp with a power of 40 W and a wavelength of 240 nm, to ensure sterilization. Then, the syringe was placed in the dispenser of the 3D printer and a stainless-steel tip with a length of 6 mm and a diameter of 330 microns was used as a nozzle. A 120 mm sterile plastic Petri dish filled with distilled water was placed on the 3D-printer table. Then, 3D printing was performed.

The printed scaffolds were kept in excess distilled water for 24 h. The samples were then washed with 95% ethyl alcohol and dried at room temperature in air for 24 h.

### 4.3. Mechanical Testing

**Figure 12:**
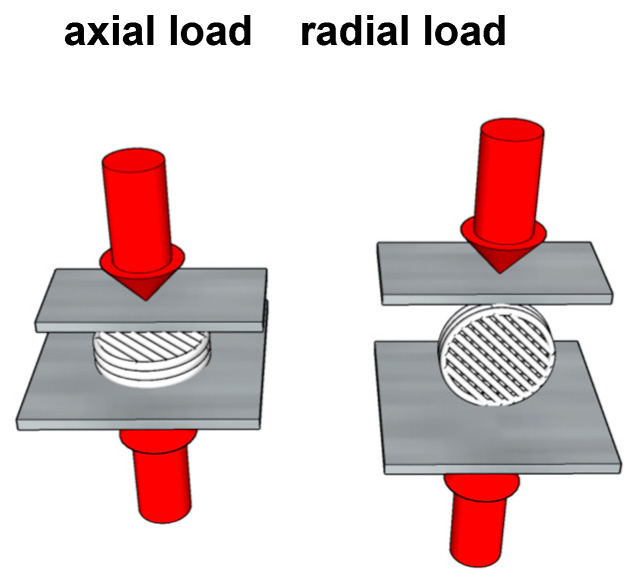
PLGA scaffold mechanical test load scheme.

Analysis of the mechanical properties of the specimens was carried out using an EZ-Test EZ-SX testing machine (Shimadzu, Kyoto, Japan). The mechanical properties were investigated using compression tests. The scaffold specimens were fixed with acrylic adhesive between testing machine plates to provide radial or axial loading (Figure 12). TRAPEZIUM X v.1.3.1 software (Shimadzu, Kyoto, Japan) was used to control the operation of the testing machine, the selection and setting of the compression test parameters (at a speed of 1 mm/min with no specific test limits), and the determination of Young’s modulus values. The Young’s moduli of the specimens were calculated from the linear regions of the stress–strain curves. The mechanical properties of each type of load direction were tested on a minimum of 5 specimens to obtain the statistics.

### 4.4. Production of Adenoviral Vectors

Adenoviral vectors encoding the BMP2 gene were synthetized according to prescribed technology. There were produced in HEK293 cells (ATCC, Manassas, VA, USA). The HEK293 cells were cultivated until they reached approximately 80% confluence and were subsequently incubated with a suspension of viral particles in a medium supplemented with 2% fetal bovine serum (FBS) for a duration of 48 h. After incubation, the cells were lysed through a freeze–thaw protocol involving three cycles of freezing at −80 °C and thawing at 37 °C. The resulting suspension of adenoviral particles was centrifugated at 1500 rpm to eliminate cellular debris and subsequently filtered through 0.22 μm diameter syringe filters (GVS, Sanford, ME, USA). Viral DNA was isolated using the QIAamp MinElute Virus Spin Kit (QIAGEN, Hilden, Germany) in accordance with the manufacturer’s protocol. The concentration of the isolated DNA was determined using a NanoDrop OneC spectrophotometer (Thermo Fisher Scientific, Waltham, MA, USA) at an absorbance wavelength of 260 nm.

### 4.5. Impregnation of Adenoviral Vectors

Adenoviral constructs with BMP2 gene were impregnated by incubation of PLGA-based 3D scaffolds with Ad-BMP2 suspension with viral DNA concentration of 125 ng/μL for 24 h.

### 4.6. Experimental Groups

We investigated 3D-printed scaffolds based on PLGA that were infused with adenoviral constructs carrying the BMP2 gene (Ad-BMP2). To assess the specific effects, we included control groups consisting of pure PLGA scaffolds devoid of adenoviral constructs, as well as wells without cells or defects containing no material (Table 1). This methodological design allowed for the isolation of matrix-related influences on the transduction process.

### 4.7. Scanning Electron Microscopy

The macro- and microstructural features of the 3D scaffolds and the morphology of their surfaces were examined by scanning electron microscopy (SEM) (Phenom ProX microscope, Eindhoven, The Netherlands). The acceleration voltage used to obtain SEM images was 15 kV. The samples were mounted on the microscope stage using conductive carbon tape.

### 4.8. Cell Culture

Multipotent mesenchymal stromal cells obtained from the adipose tissue of rats (ADSC) or HEK293 were cultured in a basic medium: DMEM/F12 (PanEco, Moscow, Russia), 10% FBS (BioSera, Cholet, France), 0.584 mg/mL L-glutamine (PanEco, Moscow, Russia) and antibiotic (5000 units/mL penicillin and 5000 µg/mL streptomycin) (PanEco, Moscow, Russia) in Petri dishes. Cells were cultured at 37 °C in an atmosphere of 5% CO_2_. The growth medium was changed every 3 days.

### 4.9. Biocompatibility

Prior to the experiment, the investigated scaffolds were sterilized through immersion in 70% ethanol for two consecutive cycles of 10 min, followed by rinsing in a physiological saline solution. To evaluate the cytocompatibility of the obtained 3D-printed PLGA-based scaffolds, ADSCs were seeded in the wells of a 24-well plate equipped with a Transwell system featuring 8 μm pores (SPL Lifesciences, Naechon-Myeon, Republic of Korea). After a period of 24 h, the scaffolds were placed in the upper inserts and incubated in a growth medium for a duration of 14 days. At the end of the experiment, cell viability was assessed using the MTT assay.

An analysis of cell adhesion was conducted using PKH-26 staining (red fluorescent cell linker kit, Sigma-Aldrich, St. Louis, MO, USA). The 3D-printed PLGA scaffolds were placed at the bottom of the wells in the 24-well plates, and a cell suspension—previously stained with the vital membrane red dye PKH-26 in accordance with the manufacturer’s protocol—was seeded onto the surface of the scaffolds and incubated for 1 and 14 days. At the end of the experiment, live cells were identified through staining with the vital dye Calcein AM at a concentration of 0.5 µM (Biotium, Fremont, CA, USA) for 35 min at 37 °C. To detect apoptotic cells, a fluorescent dye DAPI (4,6-diamidino-2-phenylindole) at a concentration of 1 µg/mL was used for 10 min. Fluorescence microscopy was performed using a Lionheart FX automated microscope (Agilent BioTek, Santa Clara, CA, USA).

### 4.10. In Vivo Study

The osteoinductive properties of the scaffolds were evaluated in vivo utilizing a critical-size calvarial bone defect model. The study was conducted on Wistar rats weighing 250–300 g. Each experimental group included six animals. All procedures were approved by the local bioethics committee of Sechenov University (Protocol No. PRC-079, dated 6 April 2024). All procedures were performed in accordance with the Guidelines for the Care and Use of Laboratory Animals established by the US National Institutes of Health (NIH Publication No. 85-23, revised 1996), the European Convention for the Protection of Vertebrate Animals used for Experimental and Other Scientific Purposes, and ISO 10993-2:2022 [32]. 

The animals were anesthetized via intraperitoneal injection of Zoletil (Virbac, France) and Xylazine (Interchemie Werken ‘de Adelaar’ BV, Castenray, The Netherlands) at doses of 30 mg/kg and 5 mg/kg, respectively. A 7 mm diameter cranial bone defect was created using a C-reamer trepan (Neobiotech, LZQ, Seoul, Republic of Korea), with continuous irrigation using sterile physiological saline solution. The scaffolds were subsequently implanted into the bone defect, and the skin was sewn up using 5/0 Vicryl sutures.

Eight weeks post-surgery, the animals were humanely euthanized via CO_2_ inhalation. The obtained biopsy specimens from the implantation site of the scaffolds were fixed in a 10% neutral formalin solution (Labico, St. Petersburg, Russia) for at least 24 h prior to histological examination.

### 4.11. Histological Study

For bone necropsies, decalcification was performed in Trilon B (Biovitrum, Saint Petersburg, Russia) for 14 days according to the generally accepted method. After dehydration in a gradient of alcohols and xylene, the specimens were embedded in paraffin. Sections of 5–10 μm thickness were then cut. The sections were stained with hematoxylin and eosin and Masson’s trichrome with aniline blue(Biovitrum, Saint Petersburg, Russia).

### 4.12. Immunohistochemical Study

Immunohistochemical (IHC) staining was used to confirm osteogenic differentiation under the action of Ad-BMP-2. Rabbit polyclonal antibodies against alkaline phosphatase (ALPL) and osteopontin (OPN) (ab95462, ab8448, Abcam, Waltham, MA, USA) were used with a DAB-based imaging system (GTX73338) followed by hematoxylin staining. IHC was performed according to the manufacturer’s protocol (GeneTex, Irvine, CA, USA).

### 4.13. Morphometry

To construct panoramas of the whole histological section, we used a software and hardware complex from Carl Zeiss based on ZEN v3.0 and the Lab.A1 light microscope (Oberkochen, Baden-Württemberg, Germany). To estimate the ratio of the area of newly formed bone (Nb.Ar.%) to the defect area, the images were segmented in Gimp v2.10 (lic. GPL-3) and the measurement of the defect and bone tissue area was performed in ImageJ v2.16 (lic. BSD-2).

### 4.14. Statistics

Statistical analysis was conducted using the GraphPad Prism software v.10.3 (GraphPad Software, Inc., San Diego, CA, USA). Data were presented as the mean ± SD (standard deviation). Normality of distribution was tested by using the Shapiro–Wilk test. Intergroup comparisons were performed using Student’s *t*-test for comparisons of two groups or the Bonferroni test for multiple group comparisons.

## Figures and Tables

**Table 1:** Experimental groups.

Group Name	Description
Control	Without scaffold (empty)
PLGA	3D-printed polylactide-co-glycolic acid scaffolds
PLGA+Ad-BMP2	3D-printed polylactide-co- glycolic acid scaffolds impregnated with adenoviral vectors with the target gene BMP2

## Data Availability

The data will be available to researchers on request.
